# The genome sequence of a metallic wood-boring beetle, *Agrilus cyanescens *(Ratzeburg, 1837)

**DOI:** 10.12688/wellcomeopenres.20877.1

**Published:** 2024-02-19

**Authors:** Mark G. Telfer, Dominic Phillips, University of Oxford and Wytham Woods Genome Acquisition Lab, Darwin Tree of Life Barcoding collective, Wellcome Sanger Institute Tree of Life Management, Samples and Laboratory team, Wellcome Sanger Institute Scientific Operations: Sequencing Operations, Wellcome Sanger Institute Tree of Life Core Informatics team, Tree of Life Core Informatics collective, Darwin Tree of Life Consortium

**Affiliations:** 1 Independent researcher, Ventnor, Isle of Wight, UK; 2 Natural History Museum, London, England, UK

**Keywords:** Agrilus cyanescens, metallic wood-boring beetle, genome sequence, chromosomal, Coleoptera

## Abstract

We present a genome assembly from an individual female *Agrilus cyanescens* (metallic wood-boring beetle; Arthropoda; Insecta; Coleoptera; Buprestidae). The genome sequence is 292.3 megabases in span. Most of the assembly is scaffolded into 10 chromosomal pseudomolecules, including the X sex chromosome. The mitochondrial genome has also been assembled and is 15.91 kilobases in length.

## Species taxonomy

Eukaryota; Opisthokonta; Metazoa; Eumetazoa; Bilateria; Protostomia; Ecdysozoa; Panarthropoda; Arthropoda; Mandibulata; Pancrustacea; Hexapoda; Insecta; Dicondylia; Pterygota; Neoptera; Endopterygota; Coleoptera; Polyphaga; Elateriformia; Buprestoidea; Buprestidae; Agrilinae; *Agrilus*; *Agrilus cyanescens* (Ratzeburg, 1837) (NCBI:txid1586972).

## Background

*Agrilus cyanescens* Ratzeburg, 1837, is a metallic wood-boring beetle from the family Buprestidae, or jewel beetles. Like other species in the *Agrilus* genus, *cyanescens* can be distinguished from other Buprestids by possessing paired toothed tarsal claws, a marginal groove in the abdominal sternites and its hind 1 tarsomere measuring longer than the 2 and 3 together ( Duff & Schmidt, 2020). *A. cyanescens* can be distinguished from other members of its genus by its possession of frons with a deep longitudinal furrow and a pronotum without keels near the hind angles, *A. cyanescens* measures between 4.5–7 mm and is metallic blue in colouration ( Duff & Schmidt, 2020; Hodge, 2010). This species also displays a unique form of sexual dimorphism in comparison to other *Agrilus* species: the male *A. cyanescens* is characterised by a more deeply emarginated and robust prosternal lobe, as well as sharply protruding metacoxal plates ( Hodge, 2010).

*Agrilus cyanescens* is distributed from southwestern China across to Turkey and through to Syria, it is also found west through to France and then reaches to western Iberia ( Niehuis, 2004). *A. cyanescens* has also been recorded in Luxembourg, regionally in Italy, northwards to Denmark but is excluded in Scandinavia – in Germany it can be found throughout and widespread in lower mountain areas ( Niehuis, 2004). *A. cyanescens* is a recent species to the UK, first recorded in South Essex/Eastern London in 2008, it has also been recorded in Cambridgeshire and Hertfordshire ( Jendek & Grebennikov, 2009). Other species of *Agrilus* were fairly widespread in the UK pre-1980, however, since then records show higher populations around the London/Essex area spreading southwards to the coast and Devon, with some records spreading to eastern Wales and across to Norfolk ( Alexander, 2003). The apparent reduction in recording sites for these species has been attributed to loss in host plant species and reductions/fragmentations in habitat ( Brown *et al.*, 2015), thus, although a new arrival to the UK, the range of *A. cyanescens* may also become limited for similar reasons, highlighting the importance of gathering species records and gaining full barcode and genome records to help monitor populations of *Agrilus* in the UK.

*Agrilus cyanescens* resides in deciduous and lowland forests, occasionally at high altitudes where windbreak basins are present, but has been recorded in areas with high honeysuckle density and occasionally on motorway verges ( Niehuis, 2004). *A. cyanescens* is a polyphagous species, recorded as feeding on plants from *Quercus*, *Fagus*, *Fraxinus*, *Alnus*, *Betula, Acer*, *Ulmus*, *Castanea*, *Salix*, *Populus* and Lonicera genera ( Niehuis, 2004). Eggs of this species are singly laid in crevices in the bark of trees around 15–30 cm above soil level. The larva bore gaps into wood before pupation, creating a hook-shaped pupa in the sapwood layer ( Niehuis, 2004). Pupal development lasts one year, with adults recorded in flight usually from June to July but have also been sighted from August to September ( Niehuis, 2004).

The genome of *Agrilus cyanescens* was sequenced as part of the Darwin Tree of Life Project, a collaborative effort to sequence all named eukaryotic species in the Atlantic Archipelago of Britain and Ireland. Here we present a chromosomally complete genome sequence for *Agrilus cyanescens*, based on one specimen collected from Wytham Woods, Oxfordshire. The publication of the first genus-wide DNA reference library of Holarctic *Agrilus* ( Kelnarova *et al.*, 2019) enabled the Darwin Tree of Life Project to successfully barcode match *A. cyanescens* to the barcodes available in this library.

## Genome sequence report

**Figure 1.**
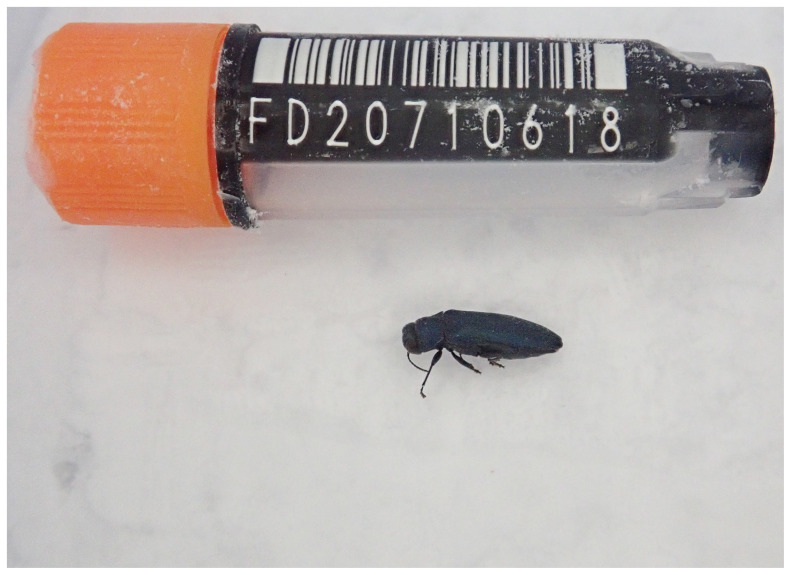
Photograph of the *Agrilus cyanescens* (icAgrCyan1) specimen used for genome sequencing.

The genome was sequenced from one female *Agrilus cyanescens* ( Figure 1) collected from Wytham Woods, Oxfordshire, UK (51.77, –1.34). A total of 56-fold coverage in Pacific Biosciences single-molecule HiFi long reads was generated. Primary assembly contigs were scaffolded with chromosome conformation Hi-C data. Manual assembly curation corrected 45 missing joins or mis-joins and removed 1 haplotypic duplications, reducing the scaffold number by 75.00%, and increasing the scaffold N50 by 109.22%.

**Figure 2.**
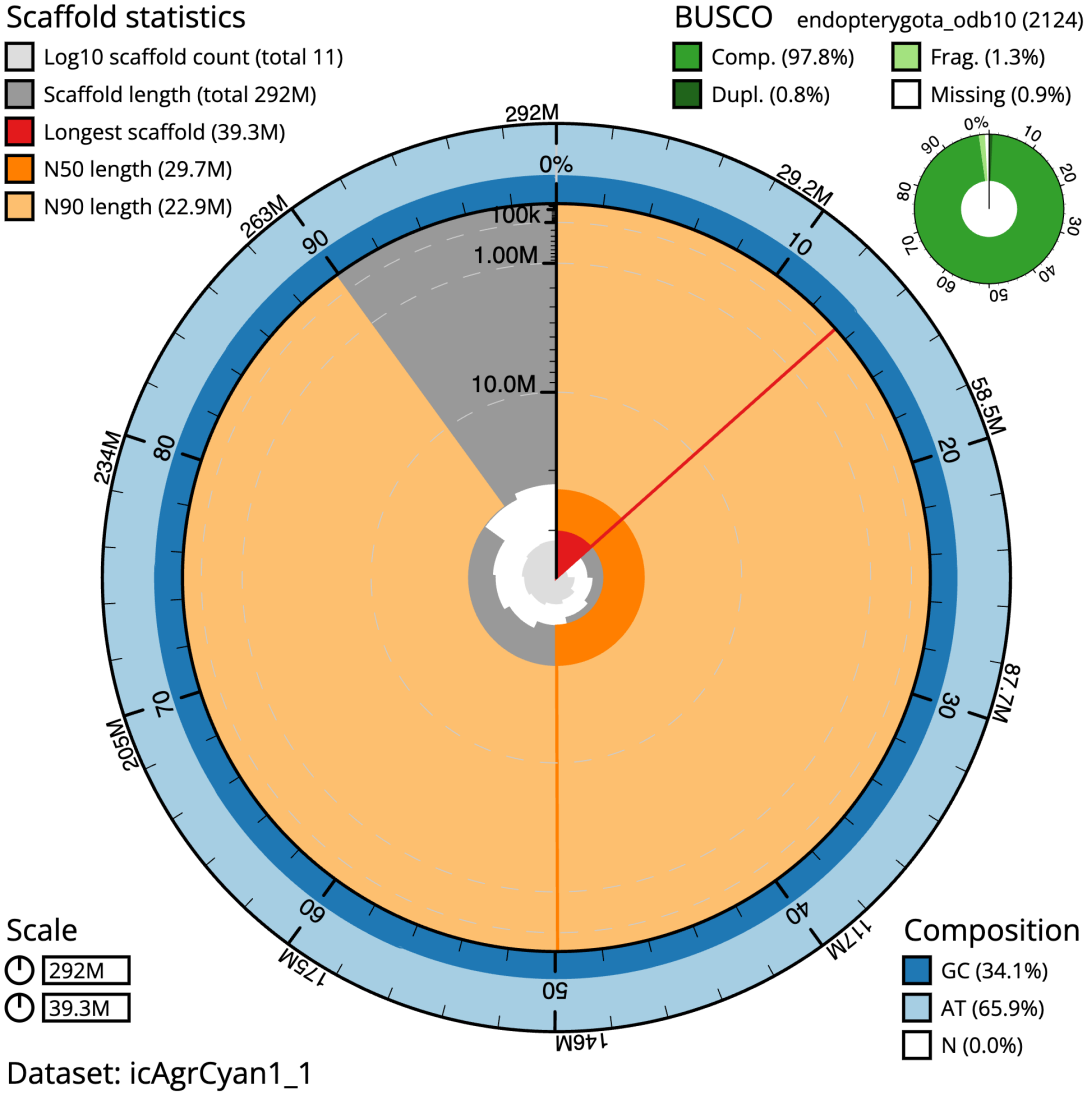
Genome assembly of *Agrilus cyanescens*, icAgrCyan1.1: metrics. The BlobToolKit Snailplot shows N50 metrics and BUSCO gene completeness. The main plot is divided into 1,000 size-ordered bins around the circumference with each bin representing 0.1% of the 292,354,128 bp assembly. The distribution of scaffold lengths is shown in dark grey with the plot radius scaled to the longest scaffold present in the assembly (39,296,097 bp, shown in red). Orange and pale-orange arcs show the N50 and N90 scaffold lengths (29,744,825 and 22,923,229 bp), respectively. The pale grey spiral shows the cumulative scaffold count on a log scale with white scale lines showing successive orders of magnitude. The blue and pale-blue area around the outside of the plot shows the distribution of GC, AT and N percentages in the same bins as the inner plot. A summary of complete, fragmented, duplicated and missing BUSCO genes in the endopterygota_odb10 set is shown in the top right. An interactive version of this figure is available at https://blobtoolkit.genomehubs.org/view/icAgrCyan1_1/dataset/icAgrCyan1_1/snail.

**Figure 3.**
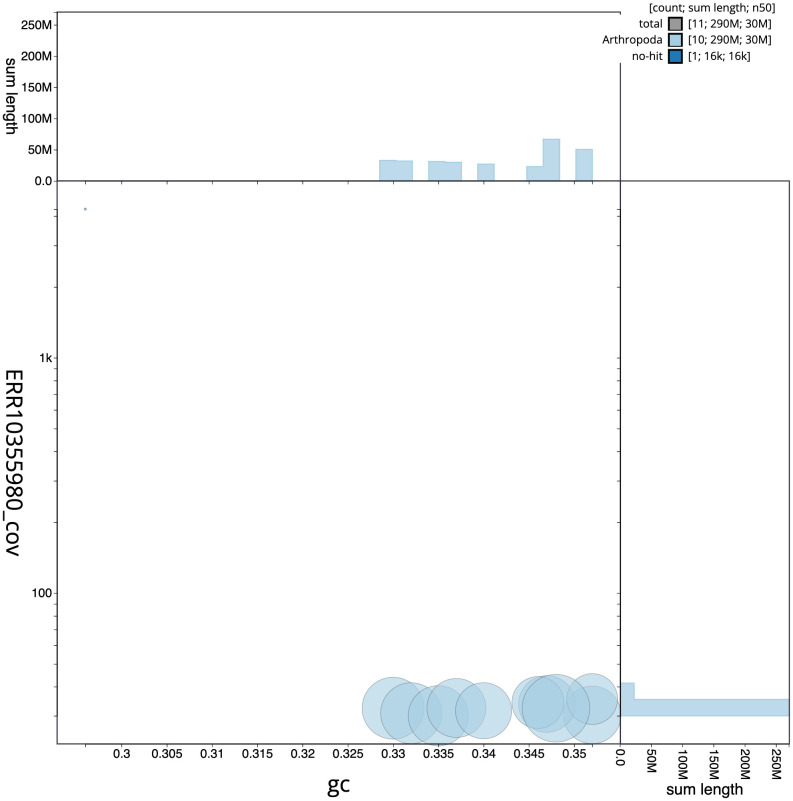
Genome assembly of *Agrilus cyanescens*, icAgrCyan1.1: BlobToolKit GC-coverage plot. Scaffolds are coloured by phylum. Circles are sized in proportion to scaffold length. Histograms show the distribution of scaffold length sum along each axis. An interactive version of this figure is available at https://blobtoolkit.genomehubs.org/view/icAgrCyan1_1/dataset/icAgrCyan1_1/blob.

**Figure 4.**
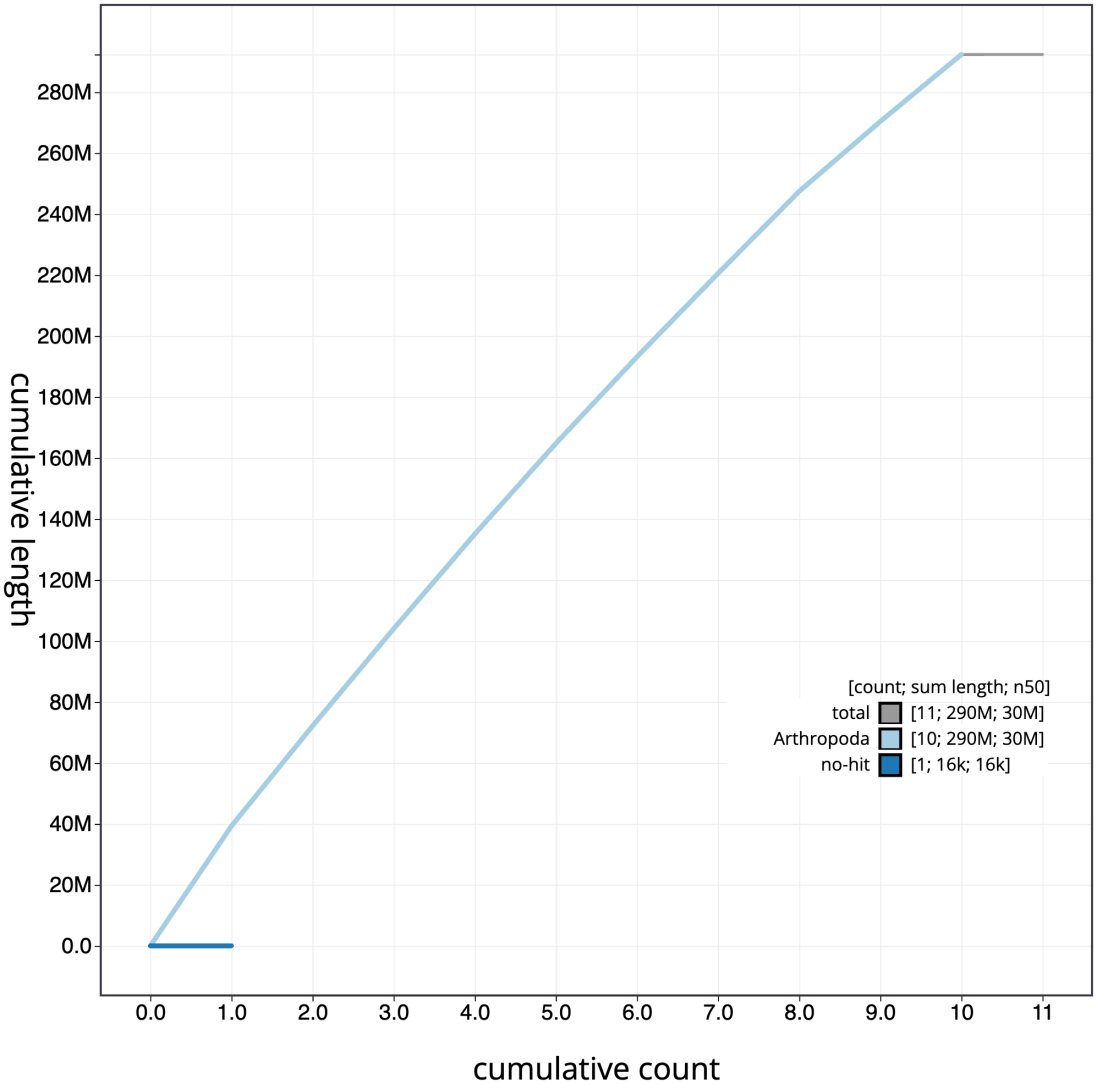
Genome assembly of *Agrilus cyanescens*, icAgrCyan1.1: BlobToolKit cumulative sequence plot. The grey line shows cumulative length for all scaffolds. Coloured lines show cumulative lengths of scaffolds assigned to each phylum using the buscogenes taxrule. An interactive version of this figure is available at https://blobtoolkit.genomehubs.org/view/icAgrCyan1_1/dataset/icAgrCyan1_1/cumulative.

**Figure 5.**
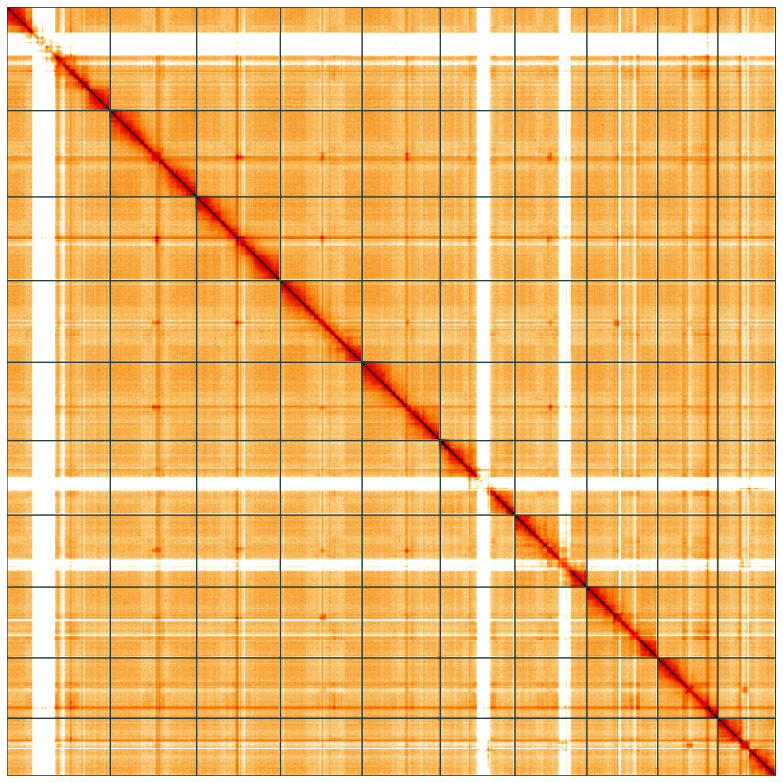
Genome assembly of *Agrilus cyanescens*, icAgrCyan1.1: Hi-C contact map of the icAgrCyan1.1 assembly, visualised using HiGlass. Chromosomes are shown in order of size from left to right and top to bottom. An interactive version of this figure may be viewed at https://genome-note-higlass.tol.sanger.ac.uk/l/?d=fNGco5NHRcqa3R73MKwNDA.

The final assembly has a total length of 292.3 Mb in 10 sequence scaffolds with a scaffold N50 of 29.7 Mb ( Table 1). The snailplot in Figure 2 provides a summary of the assembly statistics, while the distribution of assembly scaffolds on GC proportion and coverage is shown in Figure 3. The cumulative assembly plot in Figure 4 shows curves for subsets of scaffolds assigned to different phyla. Most (99.99%) of the assembly sequence was assigned to 10 chromosomal-level scaffolds, representing 10 autosomes and the XX sex chromosome. Chromosome-scale scaffolds confirmed by the Hi-C data are named in order of size ( Figure 5; Table 2). While not fully phased, the assembly deposited is of one haplotype. Contigs corresponding to the second haplotype have also been deposited. The mitochondrial genome was also assembled and can be found as a contig within the multifasta file of the genome submission.

**Table 1.** Genome data for *Agrilus cyanescens*, icAgrCyan1.1.

Project accession data		
Assembly identifier	icAgrCyan1.1	
Species	*Agrilus cyanescens*	
Specimen	icAgrCyan1	
NCBI taxonomy ID	1586972	
BioProject	PRJEB56362	
BioSample ID	SAMEA10979039	
Isolate information	icAgrCyan1 icAgrCyan1	
Assembly metrics *		*Benchmark*
Consensus quality (QV)	64.4	*≥ 50*
*k*-mer completeness	100.0%	*≥ 95%*
BUSCO **	C:97.8%[S:97.0%,D:0.8%],F:1.3%,M:0.9%,n:2,124	*C ≥ 95%*
Percentage of assembly mapped to chromosomes	99.99%	*≥ 95%*
Sex chromosomes	XX	*localised homologous pairs*
Organelles	Mitochondrial genome: 15.91 kb	*complete single alleles*
Raw data accessions		
PacificBiosciences SEQUEL II	ERR10355980	
Hi-C Illumina	ERR10313048	
Genome assembly		
Assembly accession	GCA_947389935.1	
*Accession of alternate haplotype*	GCA_947389945.1	
Span (Mb)	292.3	
Number of contigs	106	
Contig N50 length (Mb)	5.3	
Number of scaffolds	10	
Scaffold N50 length (Mb)	29.7	
Longest scaffold (Mb)	39.3	

* Assembly metric benchmarks are adapted from column VGP-2020 of “Table 1: Proposed standards and metrics for defining genome assembly quality” from ( Rhie *et al.*, 2021). ** BUSCO scores based on the endopterygota_odb10 BUSCO set using version 5.3.2. C = complete [S = single copy, D = duplicated], F = fragmented, M = missing, n = number of orthologues in comparison. A full set of BUSCO scores is available at https://blobtoolkit.genomehubs.org/view/icAgrCyan1_1/dataset/icAgrCyan1_1/busco.

**Table 2.** Chromosomal pseudomolecules in the genome assembly of *Agrilus cyanescens*, icAgrCyan1.

INSDC accession	Chromosome	Length (Mb)	GC%
OX376705.1	1	32.84	33.0
OX376706.1	2	31.86	33.0
OX376707.1	3	31.04	33.5
OX376708.1	4	29.74	33.5
OX376709.1	5	28.4	35.0
OX376710.1	6	27.37	34.5
OX376711.1	7	26.9	34.0
OX376712.1	8	22.92	34.5
OX376713.1	9	21.96	35.0
OX376704.1	X	39.3	35.0
OX376714.1	MT	0.02	29.5

The estimated Quality Value (QV) of the final assembly is 64.4 with *k*-mer completeness of 100.0%, and the assembly has a BUSCO v5.3.2 completeness of 97.8% (single = 97.0%, duplicated = 0.8%), using the endopterygota_odb10 reference set ( *n* = 2,124).

Metadata for specimens, barcode results, spectra estimates, sequencing runs, contaminants and pre-curation assembly statistics are given at https://links.tol.sanger.ac.uk/species/1586972.

## Methods

### Sample acquisition and nucleic acid extraction

A female *Agrilus cyanescens* (specimen ID Ox001780, ToLID icAgrCyan1) was potted in Wytham Woods, Oxfordshire (biological vice-county Berkshire), UK (latitude 51.77, longitude –1.34) on 2021-07-08. The specimen was collected and identified by Mark Telfer (independent entomological consultant) and preserved on dry ice.

Protocols developed by the Wellcome Sanger Institute (WSI) Tree of Life core laboratory have been deposited on protocols.io ( Denton *et al.*, 2023b). The workflow for high molecular weight (HMW) DNA extraction at the WSI includes a sequence of core procedures: sample preparation; sample homogenisation, DNA extraction, fragmentation, and clean-up. In sample preparation, the icAgrCyan1 sample was weighed and dissected on dry ice ( Jay *et al.*, 2023). Tissue from the whole organism was homogenised using a PowerMasher II tissue disruptor ( Denton *et al.*, 2023a). HMW DNA was extracted in the WSI Scientific Operations core using the Automated MagAttract v2 protocol ( Oatley *et al.*, 2023). HMW DNA was sheared into an average fragment size of 12–20 kb in a Megaruptor 3 system with speed setting 31 ( Bates *et al.*, 2023). Sheared DNA was purified by solid-phase reversible immobilisation ( Strickland *et al.*, 2023): in brief, the method employs a 1.8X ratio of AMPure PB beads to sample to eliminate shorter fragments and concentrate the DNA. The concentration of the sheared and purified DNA was assessed using a Nanodrop spectrophotometer and Qubit Fluorometer and Qubit dsDNA High Sensitivity Assay kit. Fragment size distribution was evaluated by running the sample on the FemtoPulse system.

### Sequencing

Pacific Biosciences HiFi circular consensus DNA sequencing libraries were constructed according to the manufacturers’ instructions. DNA sequencing was performed by the Scientific Operations core at the WSI on a Pacific Biosciences SEQUEL II instruments. Hi-C data were also generated from remaining tissue of icAgrCyan1 using the Arima2 kit and sequenced on the Illumina NovaSeq 6000 instrument.

### Genome assembly, curation and evaluation

Assembly was carried out with Hifiasm ( Cheng *et al.*, 2021) and haplotypic duplication was identified and removed with purge_dups ( Guan *et al.*, 2020). The assembly was then scaffolded with Hi-C data ( Rao *et al.*, 2014) using YaHS ( Zhou *et al.*, 2023). The assembly was checked for contamination and corrected using the gEVAL system ( Chow *et al.*, 2016) as described previously ( Howe *et al.*, 2021). Manual curation was performed using gEVAL, HiGlass ( Kerpedjiev *et al.*, 2018) and Pretext ( Harry, 2022). The mitochondrial genome was assembled using MitoHiFi ( Uliano-Silva *et al.*, 2023), which runs MitoFinder ( Allio *et al.*, 2020) or MITOS ( Bernt *et al.*, 2013) and uses these annotations to select the final mitochondrial contig and to ensure the general quality of the sequence.

A Hi-C map for the final assembly was produced using bwa-mem2 ( Vasimuddin *et al.*, 2019) in the Cooler file format ( Abdennur & Mirny, 2020). To assess the assembly metrics, the *k*-mer completeness and QV consensus quality values were calculated in Merqury ( Rhie *et al.*, 2020). This work was done using Nextflow ( Di Tommaso *et al.*, 2017) DSL2 pipelines “sanger-tol/readmapping” ( Surana *et al.*, 2023a) and “sanger-tol/genomenote” ( Surana *et al.*, 2023b). The genome was analysed within the BlobToolKit environment ( Challis *et al.*, 2020) and BUSCO scores ( Manni *et al.*, 2021; Simão *et al.*, 2015) were calculated.

Table 3 contains a list of relevant software tool versions and sources.

**Table 3.** Software tools: versions and sources.

Software tool	Version	Source
BlobToolKit	4.1.7	https://github.com/blobtoolkit/blobtoolkit
BUSCO	5.3.2	https://gitlab.com/ezlab/busco
gEVAL	N/A	https://geval.org.uk/
Hifiasm	0.16.1-r375	https://github.com/chhylp123/hifiasm
HiGlass	1.11.6	https://github.com/higlass/higlass
Merqury	MerquryFK	https://github.com/thegenemyers/MERQURY.FK
MitoHiFi	2	https://github.com/marcelauliano/MitoHiFi
PretextView	0.2	https://github.com/wtsi-hpag/PretextView
purge_dups	1.2.3	https://github.com/dfguan/purge_dups
sanger-tol/genomenote	v1.0	https://github.com/sanger-tol/genomenote
sanger-tol/readmapping	1.1.0	https://github.com/sanger-tol/readmapping/tree/1.1.0
YaHS	yahs-1.1.91eebc2	https://github.com/c-zhou/yahs

### Wellcome Sanger Institute – Legal and Governance

The materials that have contributed to this genome note have been supplied by a Darwin Tree of Life Partner. The submission of materials by a Darwin Tree of Life Partner is subject to the **‘Darwin Tree of Life Project Sampling Code of Practice’**, which can be found in full on the Darwin Tree of Life website here. By agreeing with and signing up to the Sampling Code of Practice, the Darwin Tree of Life Partner agrees they will meet the legal and ethical requirements and standards set out within this document in respect of all samples acquired for, and supplied to, the Darwin Tree of Life Project. 

Further, the Wellcome Sanger Institute employs a process whereby due diligence is carried out proportionate to the nature of the materials themselves, and the circumstances under which they have been/are to be collected and provided for use. The purpose of this is to address and mitigate any potential legal and/or ethical implications of receipt and use of the materials as part of the research project, and to ensure that in doing so we align with best practice wherever possible. The overarching areas of consideration are:

• Ethical review of provenance and sourcing of the material

• Legality of collection, transfer and use (national and international) 

Each transfer of samples is further undertaken according to a Research Collaboration Agreement or Material Transfer Agreement entered into by the Darwin Tree of Life Partner, Genome Research Limited (operating as the Wellcome Sanger Institute), and in some circumstances other Darwin Tree of Life collaborators.

## Data Availability

European Nucleotide Archive: *Agrilus cyanescens*. Accession number PRJEB56362; https://identifiers.org/ena.embl/PRJEB56362 ( Wellcome Sanger Institute, 2022). The genome sequence is released openly for reuse. The *Agrilus cyanescens* genome sequencing initiative is part of the Darwin Tree of Life (DToL) project. All raw sequence data and the assembly have been deposited in INSDC databases. The genome will be annotated using available RNA-Seq data and presented through the Ensembl pipeline at the European Bioinformatics Institute. Raw data and assembly accession identifiers are reported in Table 1.

## Author information

Members of the University of Oxford and Wytham Woods Genome Acquisition Lab are listed here: https://doi.org/10.5281/zenodo.7125292.

Members of the Darwin Tree of Life Barcoding collective are listed here: https://doi.org/10.5281/zenodo.4893703.

Members of the Wellcome Sanger Institute Tree of Life Management, Samples and Laboratory team are listed here: https://doi.org/10.5281/zenodo.10066175.

Members of Wellcome Sanger Institute Scientific Operations: Sequencing Operations are listed here: https://doi.org/10.5281/zenodo.10043364.

Members of the Wellcome Sanger Institute Tree of Life Core Informatics team are listed here: https://doi.org/10.5281/zenodo.10066637.

Members of the Tree of Life Core Informatics collective are listed here: https://doi.org/10.5281/zenodo.5013541.

Members of the Darwin Tree of Life Consortium are listed here: https://doi.org/10.5281/zenodo.4783558.
