# Supplementary material for: The genome sequence of a metallic wood-boring beetle, Agrilus cyanescens (Ratzeburg, 1837)
Source: Wellcome Open Res. 2024 Oct 17;9:46. Originally published 2024 Feb 19. [Version 2] doi: 10.12688/wellcomeopenres.20877.2 (PMC11109541; doi:10.12688/wellcomeopenres.20877.2)
Supplement: Peer review history

# Reviewer report: Reviewer response for version 2

**DOI:** 10.21956/wellcomeopenres.25632.r105777

**Published:** 2024-10-18

**Authors:** Will Nash

**Affiliations:** 1 Norwich Research Park, Earlham Institute, Norwich, England, UK

I am most grateful to the Team for addressing the comments in my previous reveiew.

 I am glad to see a more detailed version of the Hi-C methods used in this study.

Are sufficient details of methods and materials provided to allow replication by others?

No

Is the rationale for creating the dataset(s) clearly described?

Yes

Are the datasets clearly presented in a useable and accessible format?

Yes

Are the protocols appropriate and is the work technically sound?

Yes

Reviewer Expertise:

Evolutionary Biology, Comparative Genomics,  Population Genomics, Bioinformatics, Genome Assembly

I confirm that I have read this submission and believe that I have an appropriate level of expertise to confirm that it is of an acceptable scientific standard.

# Reviewer report: Reviewer response for version 1

**DOI:** 10.21956/wellcomeopenres.23099.r80859

**Published:** 2024-05-10

**Authors:** Will Nash

**Affiliations:** 1 Norwich Research Park, Earlham Institute, Norwich, England, UK

The authors present a chromosomal assembly of the genome of the Bupestrid beetle *A. cyanescens.*

 The introduction is well written and describes the distinguishing characters of the species, it's distribution and recording history, and some of known ecology. 

 The assembly described is produced using cutting edge techniques and seems to be of very high quality. The genome is reported at 292.3Mb in span and assembled over 10 chromosomal scaffolds. N50 is 29.7Mb, BUSCO completeness is 97.8%, QV is 64.4. 

 The methods section provides a thorough description of all collection and wet lab methods used, with the obvious exception of the Hi-C protocol used. The methods are not reproducible due to this. Hi-C is a complex and often highly species specific approach, more detail is needed to make this paper a useful resource for the community. 

 The computational methods are clear but should be more detailed - what settings are used. Again, not reproducible. 

 No annotation is provided for this species.

 Data and assembly available through ENA, genome specific accession could be included for clarity.

Are sufficient details of methods and materials provided to allow replication by others?

No

Is the rationale for creating the dataset(s) clearly described?

Yes

Are the datasets clearly presented in a useable and accessible format?

Yes

Are the protocols appropriate and is the work technically sound?

Yes

Reviewer Expertise:

Evolutionary Biology, Comparative Genomics,  Population Genomics, Bioinformatics, Genome Assembly

I confirm that I have read this submission and believe that I have an appropriate level of expertise to state that I do not consider it to be of an acceptable scientific standard, for reasons outlined above.

# Response

**Published:** 2024-05-10

**Authors:** Tree of Life Team Sanger

**Affiliations:** Wellcome Trust Sanger Institute, UK

Thank you for your comments on this data note. Please note that the Hi-C protocol is provided in the **Methods | Sequencing **section. The standard manufacturer's protocol for the Arima-HiC kit was followed.

# Reviewer report: Reviewer response for version 1

**DOI:** 10.21956/wellcomeopenres.23099.r80856

**Published:** 2024-05-10

**Authors:** Lihuén Iraí González Dominici

**Affiliations:** 1 Microbiology and Genetics Department, University of Salamanca, Salamanca, Spain

Dear all,

 I am pleased for the opportunity to review the publication titled “The genome sequence of a metallic wood-boring beetle, *Agrilus cyanescens* (Ratzeburg, 1837)”. This article presents the genome assembly for an individual female metallic wood-boring beetle. The genome assembly is 292.3 megabases in length and has been scaffolded into 10 chromosomal pseudomolecules, including X sex chromosome. The mitochondrial genome assembly is also provided.

 This manuscript is well-written, concise and uses appropriate terminology. Relevant information on the biology, morphology and phylogeny of this species is included in the background, enhancing reader experience. The methodology used is sufficiently clear and ensures the reproducibility by other researchers. The overall quality of this publication is noteworthy, providing relevant information for future studies concerning *A. cyanescens*.

 However, I have a few minor suggestions.

- Firstly, in the background, I would appreciate the inclusion of information regarding the ecological significance of this insect. It is known that wood-boring beetles play a pivotal role in many ecosystems, as they have an influence on plant-associated microbial. Do we have any insights into the specific impact of this beetle species? This could be relevant information in stimulating further research within the field of microbiology.
- Secondly, concerning Figure 5, while the order of the chromosomes is detailed clearly at the bottom of the figure, as a reader, it would help me if they were also presented in the plot.
- Finally, I propose that an annotation of the genome of these insects greatly enriches the knowledge of the important role of wood-boring beetles within ecosystems and thus increases the level of impact of this already valuable work. Naturally, this suggestion could be considered for future research.

Are sufficient details of methods and materials provided to allow replication by others?

Yes

Is the rationale for creating the dataset(s) clearly described?

Yes

Are the datasets clearly presented in a useable and accessible format?

Yes

Are the protocols appropriate and is the work technically sound?

Yes

Reviewer Expertise:

Microbiology and Bioinformatics

I confirm that I have read this submission and believe that I have an appropriate level of expertise to confirm that it is of an acceptable scientific standard.

# Reviewer report: Reviewer response for version 1

**DOI:** 10.21956/wellcomeopenres.23099.r77012

**Published:** 2024-04-25

**Authors:** Jan Veenstra

**Affiliations:** 1 INCIA, UMR 5287, CNRS, University of Bordeaux, Pessac, France

Major.

 This is yet another high quality genome assembly from the Welcome Sanger Institute. As sequencing technologies have improved, so have genome assemblies. Progress has been truly impressive. I am not a reviewer who himself produces genome assemblies, rather I am one of those who uses genome assemblies in order to help understand some aspects of insect physiology, neuropeptides in my case. So I quickly checked the various neuropeptide genes that are present in insects, and the genome seems complete. However, as a user I like genome assemblies that are not only based on Biopac sequences, even though these have also enormously improved over the years, but include illumina genomic sequences. The latter are really useful to check whether indeed sequences in the assemblies that look like they might have an indel are indeed correct. Furthermore, I really like the many-fold coverage of illumina sequences as it allows one to convince oneself that a particular gene is truly lacking from a species. Obviously, it is also very helpful if there is some transcriptome data, but that is just icing on the cake. This is the only somewhat more negative point I see with this assembly, although I assume that there simply was not enough DNA from the single individual.

 Two minor points.

 I think that if I wanted to sequence a genome from a new species, I would prefer a male, as it would include the Y chromosome. I also think this type of manuscript is probably most used, although perhaps not carefully read, by people like me who use the generated data. For us it might be useful to explain a bit what the white area in Figure 5 mean and what they imply. Does it indeed mean that those parts of the assembly are perhaps not correctly arranged ?

Are sufficient details of methods and materials provided to allow replication by others?

Yes

Is the rationale for creating the dataset(s) clearly described?

Yes

Are the datasets clearly presented in a useable and accessible format?

Yes

Are the protocols appropriate and is the work technically sound?

Yes

Reviewer Expertise:

Insect neuropeptides

I confirm that I have read this submission and believe that I have an appropriate level of expertise to confirm that it is of an acceptable scientific standard.

# Response

**Published:** 2024-10-10

**Authors:** Tree of Life Team Sanger

**Affiliations:** Wellcome Trust Sanger Institute, UK

Thank you for reviewing this data note.  We plan to provide transcriptome data for all BioProjects within the Darwin Tree of Life (DToL) programme, adding the raw data to the BioProject and this data note as it becomes available.

 While Illumina Chromium 10X technology is no longer available, PacBio HiFi reads offer high accuracy, as indicated by the QV of 64.6, which is sufficient for indel detection.

 Although sequencing the heterogametic sex is preferred, we are dependent on what collectors can obtain during fieldwork. For this project, only a single specimen was collected, which happened to be female.
